# Clinical Utility of Bronchoalveolar Lavage Pepsin in Diagnosis of Gastroesophageal Reflux among Wheezy Infants

**DOI:** 10.1155/2016/9480843

**Published:** 2016-07-19

**Authors:** Ahmed Fathi Abdallah, Tarek El-Desoky, Khalid Fathi, Wagdy Fawzi Elkashef, Ahmed Zaki

**Affiliations:** ^1^Gastroenterology and Hepatology Unit, Pediatric Department, Faculty of Medicine, Mansoura University, Mansoura 35516, Egypt; ^2^Allergy, Respiratory, and Clinical Immunology Unit, Pediatric Department, Faculty of Medicine, Mansoura University, Mansoura 35516, Egypt; ^3^Pediatric Department, Faculty of Medicine, Mansoura University, Mansoura 35516, Egypt; ^4^Pathology Department, Faculty of Medicine, Mansoura University, Mansoura 35516, Egypt

## Abstract

*Background*. There is no gold standard test for diagnosis of gastroesophageal reflux disease (GERD) associated infantile wheezing.* Objectives*. To evaluate the value of bronchoalveolar lavage (BAL) pepsin assay in diagnosis of GERD in wheezy infants.* Methods*. Fifty-two wheezy infants were evaluated for GERD using esophageal combined impedance-pH (MII-pH) monitoring, esophagogastroduodenoscopy with esophageal biopsies, and BAL pepsin. Tracheobronchial aspirates from 10 healthy infants planned for surgery without history of respiratory problems were examined for pepsin.* Results*. Wheezy infants with silent reflux and wheezy infants with typical GERD symptoms but normal MII-pH had significantly higher BAL pepsin compared to healthy control (45.3 ± 8.6 and 42.8 ± 8 versus 29 ± 2.6, *P* < 0.0001 and *P* = 0.011, resp.). BAL pepsin had sensitivity (61.7%, 72 %, and 70%) and specificity (55.5%, 52.9%, and 53%) to diagnose GERD associated infantile wheeze compared to abnormal MII-pH, reflux esophagitis, and lipid laden macrophage index, respectively.* Conclusion*. A stepwise approach for assessment of GERD in wheezy infants is advised. In those with silent reflux, a trial of antireflux therapy is warranted with no need for further pepsin assay. But when combined MII-pH is negative despite the presence of typical GERD symptoms, pepsin assay will be needed to rule out GERD related aspiration.

## 1. Introduction

Recurrent wheezing is common in children. Although almost 50 percent of children are reported to have wheezing in the first year of life, only 20 percent will experience continued wheezing symptoms in later childhood [1, 2]. Gastroesophageal reflux disease (GERD) has been incriminated as one of the several causes of nonasthmatic wheezing in young children and infants [3]. Both establishment of causality and accurate diagnosis have not been reached [4]. Diagnosis of GERD related infantile wheeze is made using both clinical background and supporting diagnostic tests [5]. However, there is no single test that is a gold standard for diagnosis [4]. Furthermore, some patients with a respiratory disease and normal GERD diagnostic tests can respond to antireflux surgery [6]. Several biomarkers have been proposed for diagnosis of GERD related airway diseases. The most common is lipid laden macrophage index (LLMI) but its accuracy in diagnosis of GERD related respiratory symptoms is questionable [7, 8].

A sensitive and specific biomarker which can explain causality and disease severity is needed. Detection of pepsin in the bronchoalveolar lavage (BAL) fluid has been proposed for diagnosis of GERD related pulmonary symptoms [9]. In this study, we aim to evaluate the value of pepsin assay in the BAL fluid in diagnosis of GERD in wheezy infants compared to the current standard diagnostic tests.

## 2. Materials and Methods

This prospective cohort study was conducted at Mansoura University Children Hospital (MUCH), Egypt, between May 2013 and May 2015 and was approved by MUCH ethical committee. An informed consent was taken from the caregivers before the study.

Fifty-two wheezy infants followed up in allergy and respiratory and clinical immunology unit at MUCH and who experienced physician documented 3 attacks of wheezing episodes over the last 6 months or persistent wheeze over the last month were evaluated for GERD at the gastroenterology unit. Wheezy infants with atopy (allergic rhinoconjunctivitis or eczema), prematurity, abnormal neurological examination, congenital heart diseases, airspace opacities on chest radiograph, tracheobronchial malformations, immune deficiency, and anatomical esophageal or gastric malformations were excluded. Additionally, tracheal aspirates from 10 healthy infants planned for surgery (8 for inguinal herniotomy and 2 for hypospadias repair) without history of any respiratory problems were obtained and examined for pepsin.

Wheezy infants were evaluated by complete history taking including symptoms suggestive of GERD (vomiting, regurgitation, choking, arching, refusal of feeding, and failure to gain weight). Combined multiple channel intraluminal esophageal impedance and pH (MII-pH) monitoring, esophagogastroduodenoscopy (EGD) with esophageal biopsies, and BAL were performed for all cases.

### 2.1. 24-Hour Combined Esophageal MII-pH Monitoring (ZepHr)

Wheezy infants underwent 24-hour combined MII-pH monitoring (ZepHr, Sandhill Scientific Inc., Highlands Ranch, CO, USA) using infant size impedance catheter (6 impedance channels, 1.5 cm distance, and esophageal pH sensor).

The pH sensor was placed opposite to the 3rd vertebral body above the diaphragmatic angle, using Strobel's mathematical model [(0.25 × height in cm) + 5] for infants younger than one year and EGD for older infants to determine the lower esophageal sphincter position. The position of the pH sensor was confirmed by CXR. Mothers were instructed to record feeding time, recumbent and upright position, and cough episodes by pressing the corresponding key on the ZepHr recorder.

The studies (for a duration of at least 20 hours) were transferred to and analyzed by ZepHr software. Reflux episode was defined as “a retrograde drop in impedance of at least 50% of the baseline in at least 2 distal impedance channels,” classified into acidic and nonacidic based on pH <4 or pH >4, respectively. Bolus exposure index was determined by the percentage of time that all boluses were present in the esophagus, while the reflux index (RI) was determined by the percentage of time with esophageal pH <4 [10]. Reflux-cough correlation was measured using symptom index (SI) [11], symptom sensitivity index (SSI) [12], and symptom association probability (SAP) [13] provided that cough episode occurred within 2 minutes of a reflux event; at least 5 symptoms were required to be validated for analysis.

Abnormal pH study was defined by RI ≥10% in infants younger than one year or ≥5% in infants older than one year [14]. According to German pediatric impedance group (G_PIG), abnormal MII study was considered when the total number of reflux episodes was ≥100 in infants younger than one year or ≥73 in infants older than one year: reflux-cough SI ≥50%, reflux-cough SSI ≥10%, or reflux-cough SAP ≥95% [15].

### 2.2. BAL

Infants underwent diagnostic rigid bronchoscope under general anesthesia. Infants were fasting prior to the procedure for 6 hours for milk and solids and 4 hours for water. BAL was performed via bronchoscope tube wedged in the right lower lobe bronchus. Three times 1 mL/kg of 0.9% saline solution was instilled into the lavage site and gently suctioned. BAL was technically accepted if ≥40% of instilled fluid was recovered. BAL fluid was centrifuged and the supernatant fluid was stored at −40°C to be examined for pepsin. Two slides were prepared from the cellular debris. 100 alveolar macrophages were tested for lipid content using Oil Red O stain and LLMI was calculated according to the standard method [16]. The second slide was stained with H&E for the evaluation of differential leukocytes counts by counting 300 leukocytes.

#### 2.2.1. Tracheobronchial Aspirate

After insertion of the endotracheal tube, the infant's head was tilted to the left side and the tube was advanced as far as possible. Three samples of 0.9% saline solution were instilled and then recovered in a manner similar to BAL technique.

#### 2.2.2. BAL Fluid Pepsin Measurement

Human Pepsin ELISA Kit of Glory Science Co., Ltd., was used to measure BAL pepsin content. The prepared samples were added to tests wells; then, both pepsin antibody (10 *μ*L) and streptavidin HRP 50 *μ*L were added. Tests wells were sealed and gently shaken and incubated for 60 minutes at 37°C. Plates were washed five times followed by addition of chromogen solution A&B and were left to react for 10 minutes at 37°C. Then, stop solution was added and the optical density value was measured within 10 minutes. The corresponding sample's concentration was calculated according to the sample's optical density.

### 2.3. Statistical Analysis

Testing was performed using SPSS 21.0. Shapiro-Wilk test was used to test distribution of data. Independent sample *t*-test, Mann-Whitney* U* test, and One-Way ANOVA with* post hoc* Tukey test or Chi-square test were used to compare parametric, nonparametric, and multiple independent samples and qualitative data, respectively. Spearman correlation coefficient was used to assess the correlation between variables. A probability value of *P* < 0.05 indicated statistical significance between groups.

## 3. Results

### 3.1. Demographic Data

Fifty-two wheezy infants were included in the study (Table 1).

### 3.2. BAL Pepsin Results

**Figure 1:**
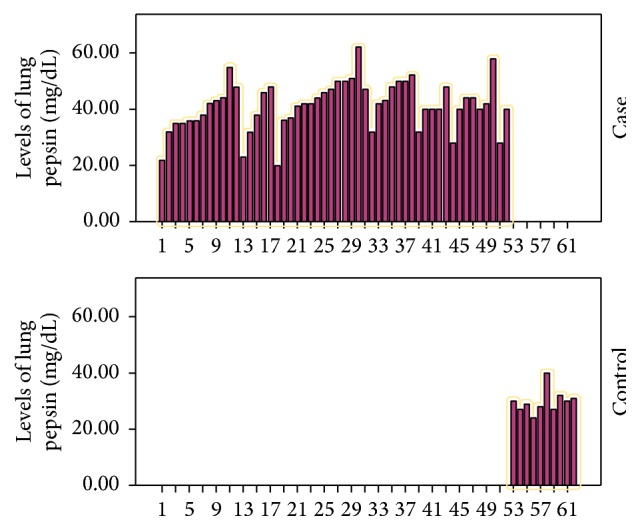
Lung pepsin levels in wheezy infants and healthy control.

BAL pepsin results are listed in Figure 1. According to the presence of typical GERD symptoms and the result of combined MII-pH, we classified wheezy infants into 4 groups (Table 2). Wheezy infants with silent reflux (group 2) and wheezy infants with typical GERD symptoms but normal combined MII-pH (group 3) had significantly higher pepsin level compared to healthy control (45.3 ± 8.6 and 42.8 ± 8 versus 29 ± 2.6, *P* < 0.0001  and  0.011, resp.). Also, wheezy infants with typical GERD symptoms and/or abnormal combined MII-pH had significantly higher pepsin level than healthy control but not than those with no symptoms and normal impedance-pH (42.1 ± 8.3 versus 29 ± 2.6 and  37.9 ± 6.8, *P* < 0.0001  and  >0.05, resp.).

### 3.3. BAL Pepsin Correlation with Clinical Data, BAL Cellular Contents, and MII-pH Parameters

BAL fluid pepsin level did not show significant correlation with age, age of onset of wheeze, body weight, peripheral eosinophilia, BAL differential leukocytes percentage, or LLMI. It had a weak significant negative correlation with median bolus clearance time in infants younger than one year (*r* = −0.321, *P* = 0.05), while in infants older than one year it had a high significant positive correlation with the mean acid clearance time and the duration of the longest acid episode (*r* = 0.745, *P* = 0.002; *r* = 0.587, *P* = 0.02, resp.) (Table 3).

### 3.4. Relation between Abnormal BAL Pepsin and Abnormal GERD Diagnostic Tests

When we assumed the highest level of pepsin obtained in healthy control (40 ng/mL) to be the upper limit of normal, 29 (55.8%) of wheezy infants had abnormal pepsin level. Nonsignificant relations were found between abnormal BAL pepsin and abnormal combined MII-pH, reflux esophagitis, or LLMI ≥ 100 (*P* > 0.05) (Table 4). Accordingly, BAL pepsin sensitivity, specificity, and positive and negative predictive values to diagnose reflux were calculated (Table 5).

## 4. Discussion

Pepsin is a proteolytic enzyme secreted by gastric chef cells that should not be detected in the lower respiratory tract. Therefore, its detection in the BAL fluid should be a highly sensitive and specific marker for aspiration [17].

In the current study, BAL pepsin was positive in wheezy infants and in the healthy control subjects. The presence of pepsin in the lung of healthy control could be explained by the finding that a significant portion of healthy individuals aspirates nasopharyngeal secretions during sleep due to drop of the upper oesophageal sphincter tone [18]. So the presence of pepsin per se in the BAL may not necessarily always be an abnormal finding. Another explanation is that multiple pepsin isoforms exist. The relationship between these isoforms and respiratory complications is unclear [19]. Unlike pepsinogen A, pepsinogen C has been identified in extragastric sites, including the lungs. Pepsinogen C is expressed by type 2 pneumocytes and is involved in surfactant B processing [20, 21].

We also documented that some wheezy infants with normal combined MII-pH monitoring had abnormal BAL pepsin level. This denotesthat dependence on esophageal MII-pH measurements may be insufficient for the assessment of GERD in clinical practice as microaspiration may occur earlier than the time of MII-pH monitoring. On the other hand, BAL pepsin was found to be normal in some wheezy infants with abnormal GERD diagnostic tests. This is explained by the fact that a diagnosis of GERD does not mean that patients are refluxing out of the esophagus and hence aspirating. Even if the refluxate reaches the upper airway, it is almost cleared by a hyperactive cough reflex [22]. This agrees with Starosta et al. who studied 96 children with chronic respiratory symptoms using two-channel 24-hour esophageal pH measurements and found that the average concentration of pepsin in BAL fluid was higher in the group of children with extensive proximal acidic RI >2% than in children with RI <2%. However, there was overlap between the groups, and some children with normal proximal pH measurements had relatively high pepsin concentrations in their BAL, whereas others with significant reflux had no pepsin in BAL [23].

In the current study, BAL pepsin showed a high significant positive correlation with the mean acid clearance time and the duration of the longest acid episode in infants older than one year. When esophageal clearance time is prolonged, the reflux material will present for longer time in the esophagus and produce laryngopharyngeal irritation and increase the risk of aspiration [24, 25]. In younger infants, the negative correlation with the median bolus clearance time is difficult to understand. However, aspiration depends on factors other than reflux per se like the protective cough reflex and the upper esophageal sphincter competence. So the longer the time needed to clear the esophagus, the more effective the cough reflex that may be generated and the upper esophageal sphincter tone that may be increased [22]. This may also explain why the number of reflux episodes did not correlate with pepsin levels. On the other hand, no significant correlations were found with the actual number of proximal reflux episodes. Also, no significant correlations were found between BAL pepsin and BAL differential leukocytes percentage or LLMI.

It is rather counterintuitive that significantly higher levels of pepsin were noted in patients who have symptom only or abnormal pH-MII only compared to controls, and yet this is not observed in patients who have both abnormal symptoms and testing. This may reflect the limitations of our reference standards: reliance on symptom gathering or point-in-time pH-MII studies. This may explain why we detected low sensitivity and specificity of pepsin in predicting pathologic reflux defined by combined MII-pH, esophageal biopsy, and LLMI. Krishnan et al. found that the sensitivity of tracheal pepsin, using pH probe as the gold standard, was 78% in children with reflux and respiratory symptoms. Using esophagitis as the gold standard, they found that the sensitivity of pepsin was 83% [8]. Rosen et al. found the sensitivity and specificity of pepsin in predicting pathologic reflux by MII-pH or reflux esophagitis were 57% and 65%, respectively [26].

Our results are closer to that observed by Roesn et al. [26] but lower than that observed by Krishnan et al. [8]. As compared with the older study, the lower sensitivity in our study may be explained by the cases selected for evaluation. We evaluated all wheezy infants with or without GERD symptoms and did not limit our study to the patients with only reflux symptoms as Krishnan et al. [8] did.

The variable sensitivity and specificity of abnormal BAL pepsin compared to the current standard test raise a question whether BAL pepsin is not a reliable marker of aspiration or whether the gold standard tools for evaluating reflux (pH probe, MII-pH, and endoscopy) upon which sensitivity analysis was based are not ideal gold standards. However, we can explain this variation by the fact that pulmonary manifestations of GERD are not only secondary to direct microaspiration but also secondary to vagally mediated hyperreactivity and a neural reflex between the esophagus and the airways [27, 28]. We also support this hypothesis by our observation that a group of wheezy infants with typical GERD symptoms had normal combined MII-pH while they had BAL pepsin level significantly higher than normal controls. This means that the current definition of abnormal MII-pH may miss the diagnosis of not only typical GERD but also atypical GERD.

Our study may be limited by comparison of BAL to tracheal aspirate pepsin. However, we performed tracheobronchial aspirate, though blindly, in a way that should get the endotracheal tube wedged into the right lower lobe bronchus. So the comparison of pepsin obtained by the two methods could measure pepsin level at distal but similar sites.

**Figure 2:**
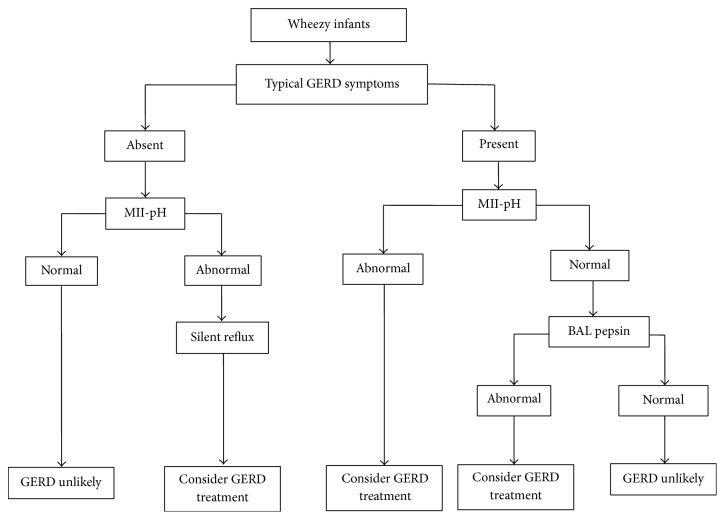
Stepwise approach for diagnosis of GERD associated wheezing in infants.

According to data obtained in our study, we suggest that BAL pepsin assay will be of diagnostic significance in a group of wheezy infants with typical GERD symptoms but normal combined MII-pH (Figure 2). In this group of patients, a trial of antireflux therapy should be carried out.

## 5. Conclusion

Nonsignificant relations between GERD standard diagnostic tests and BAL pepsin necessitate a stepwise approach for assessment of GERD in these patients. In those with silent reflux, a trial of antireflux therapy is warranted with no need for further pepsin assay. But when combined MII-pH is negative despite the presence of typical GERD symptoms, pepsin assay will be an important test to rule out GERD related aspiration.

## Figures and Tables

**Table 1:** Demographic data of the studied groups.

	Continuous wheeze group	Episodic wheeze group	*P*
*N* (%)	27 (51.9)	25 (48.1)	0.34
Age, median (rang)	9 (3–24)	9 (5–23.5)	0.79
Sex, male *N* (%)	21 (77.8)	14 (56)	0.09
Presence of typical GERD symptoms, *N* (%)	10 (37)	11 (44)	0.81
Abnormal MII-pH, *N* (%)	18 (66.6)	14 (56)	0.2
Reflux esophagitis, *N* (%)	6 (22.2)	8 (32)	0.15

GERD, gastroesophageal reflux disease; MII-pH, multiple channel intraluminal impedance-pH.

**Table 2:** BAL pepsin in wheezy infants based on the presence of GERD compared to healthy control.

	Group 1	Group 2	Group 3	Group 4	Group 5	Control	*P*
*N*	13	21	8	10	42	10	
Pepsin (mean ± SD)	36.5 ± 7.8	45.3 ± 8.6^A^	42.8 ± 8^B^	37.9 ± 6.8	42.1 ± 8.3^C^	29 ± 2.6^A,B,C^	<**0.0001**

Group 1: typical GERD symptoms present and combined MII-pH positive, Group 2: typical GERD symptoms absent and combined MII-pH positive, Group 3: typical GERD symptoms present and combined MII-pH negative, Group 4: typical GERD symptoms absent and combined MII-pH negative, and Group 5: GERD symptoms and/or combined pH-MII positive. Similar letters indicate significance between groups; A (*P* <** 0.0001**), B (*P* =** 0.011**), and C (*P* <** 0.0001**).

**Table 3:** Bronchoalveolar fluid pepsin correlation with MII-pH monitoring parameters.

BAL pepsin				
	Below one year		1-2 years	
	*r*	*P*	*r*	*P*
pH monitoring parameters				
Number of acid reflux episodes	0.03	0.858	−0.06	0.84
Reflux index	0.06	0.71	0.409	0.146
Mean acid clearance time	0.02	0.906	0.745	**0.002**
Longest acid episode	0.101	0.546	0.587	**0.02**
Acid episodes longer than 5 minutes	0.005	0.97	0.47	0.08

MII parameters				
Acid percent time	0.08	0.634	−0.033	0.244
Nonacid percent time	0.098	0.558	−0.041	0.89
Bolus exposure index	−0.055	0.741	−0.091	0.75
Median bolus clearance time	−**0.321**	**0.05**	−0.305	0.29
Longest impedance episode	0.026	0.879	−0.044	0.88
Number of distal acid reflux episodes	0.037	0.825	0.006	0.985
Number of distal nonacid reflux episodes	0.135	0.419	−0.199	0.496
Number of all distal reflux episodes	0.091	0.588	−0.088	0.764
Number of proximal acid reflux episodes	−0.029	0.863	−0.16	0.584
Number of proximal nonacid reflux number episodes	0.094	0.574	−0.149	0.612
Number of all proximal reflux episodes	0.014	0.934	−0.147	0.61

BAL pepsin correlates with mean acid clearance time and the longest acid episode.

**Table 4:** Relation between BAL pepsin and GERD diagnostic tests.

		Normal pepsin *N* (%)	Abnormal pepsin *N* (%)	*P*
		23 (44.2)	29 (55.8)	
Combined MII-pH	+ve	13 (56.5)	21 (72.4)	0.23
	−ve	10 (43.5)	8 (27.6)	

Reflux esophagitis	+ve	5 (21.7)	13 (44.8)	0.14
	−ve	18 (78.3)	16 (55.2)	

LLMI	≥100	6 (26.1)	14 (48.3)	0.15
	<100	17 (73.9)	15 (51.7)	

LLMI, lipid laden macrophage index; MII-pH, multiple intraluminal impedance-pH monitoring. No significant relation occurs between abnormal BAL pepsin and abnormal combined MII-pH, reflux esophagitis, and LLMI ≥ 100.

**Table 5:** Sensitivity and specificity of abnormal BAL pepsin levels to diagnose GERD related infantile wheeze compared to the current standard diagnostic tests.

Abnormal pepsin level	Abnormal MII-pH	Reflux esophagitis	LLMI ≥ 100
Sensitivity%	61.7	72	70
Specificity%	55.5	52.9	53
Positive predictive value%	72	44.8	48.3
Negative predictive value%	43.4	78	73.9

GERD, gastroesophageal reflux disease; LLMI, lipid laden macrophage index; MII-pH, multiple channel intraluminal impedance-pH monitoring.
